# A novel and cost-effective model to screen and treat cervical cancer and precancers at the point of care

**DOI:** 10.3389/fpubh.2025.1527172

**Published:** 2025-04-16

**Authors:** Jean M. Bouquet, Emily Morris, Isain Zapata

**Affiliations:** ^1^Department of Primary Care Medicine, Rocky Vista University College of Osteopathic Medicine, Englewood, CO, United States; ^2^Rocky Vista University College of Osteopathic Medicine, Englewood, CO, United States; ^3^Department of Biomedical Sciences, Rocky Vista University College of Osteopathic Medicine, Englewood, CO, United States; ^4^Office of Research and Scholarly Activity, Rocky Vista University, Englewood, CO, United States

**Keywords:** cervical cancer, gynecology, novel vaginal speculum, AI-assisted cervicography, canister-based cryotherapy, public health, cancer prevention

## Abstract

**Background:**

Cervical cancer has a high incidence to high mortality rate and poses an important global burden that disproportionately affects women in underdeveloped areas of the world. The World Health Organization has proposed a new and ambitious policy that aims to reduce the number of deaths from cervical cancer by 62.6 million over the next 70 years. However, there are many obstacles to the adoption of this policy and implementation in both Resource-Rich and Resource-Constrained Countries.

**Methods:**

In this perspective article, we propose a cost-effective, sustainable, and practical model that introduces a kit that may help overcome some of the existing barriers and achieve the goal to eventually eliminate cervical cancer. The kit includes a novel vaginal speculum that provides better visibility of the cervix, ease-of-use, and patient comfort; AI-assisted cervicography with a portable colposcope or Smart Phone; and canister-based cryotherapy.

**Results:**

Previous studies in Peru, Panama, Paraguay, and Kenya have demonstrated that these kits represent a novel, cost-effective, practical, accurate, and sustainable model to screen, triage, and treat cervical cancer and precancers at the point of care anywhere in the world.

**Conclusion:**

This model is a simple and inexpensive solution to some of the barriers to care for cervical health, potentially providing significant benefits by decreasing morbidity and mortality of cervical cancer without significant risk for women throughout the world.

## Introduction

Cervical cancer is a slow growing cancer that is easily prevented but has a high incidence to mortality rate at close to 50%, meaning that nearly half of women will die from cervical cancer once the diagnosis is made (1). The term “women” refers to all biologic females and gender diverse people at risk for cervical cancer. Globally, there are over 340,000 women who die from this very preventable cancer every year (1).The effects from each death on the fabric of the community are vast: in 2017, the global economic burden of cervical cancer was estimated to be $682 billion (USD) (2). In addition, cervical cancer patients are nearly two times as likely to report activity limitations and poor general health and had significantly higher PHQ-2 depression severity scores (3).

The World Health Organization (W.H.O.) created the 2030, 90-70-90 policy in 2021 that aimed to reduce the number of deaths from cervical cancer by 62.6 million over the next 70 years. This policy calls for the following goal to be met by 2030: 90% vaccination of girls against the Human Papilloma Virus (HPV; the cause of 99.7% of cervical cancers) (4), 70% of women screened twice in their lifetime, and 90% of women treated for precancerous and cancerous lesions of the cervix (5). China and other countries have incorporated the W.H.O.'s policy to eliminate cervical cancer but the 90% vaccination threshold has not been met. In 2019, the global HPV vaccination rate was only 15% (6). Importantly, cervical cancer has almost been eliminated in Australia due to aggressive HPV vaccination campaigns (7). However, there are many obstacles to adoption of this policy and implementation in other countries. Individual barriers to care include lack of knowledge and awareness, fear and embarrassment, low health literacy, competing priorities, lack of trust in the healthcare system, and personal beliefs and values. Structural and system-level barriers to cervical health include insufficient and expensive tools for screening and treatment, cost and implementation of vaccination and lack of insurance, limited access to healthcare, low-quality healthcare systems, language and cultural barriers, and stigma and discrimination (8, 9). Unless and until HPV vaccination is more universally implemented at the W.H.O.'s goal of 90% by 2030, there is a need for a cost-effective, accurate, sustainable, and practical model that provides more equitable care throughout the world. This perspective article offers a new and unique model that addresses and may help overcome some of the existing barriers and achieve the goal of the W.H.O. to eventually eliminate cervical cancer.

## A new vaginal speculum

**Figure 1:**
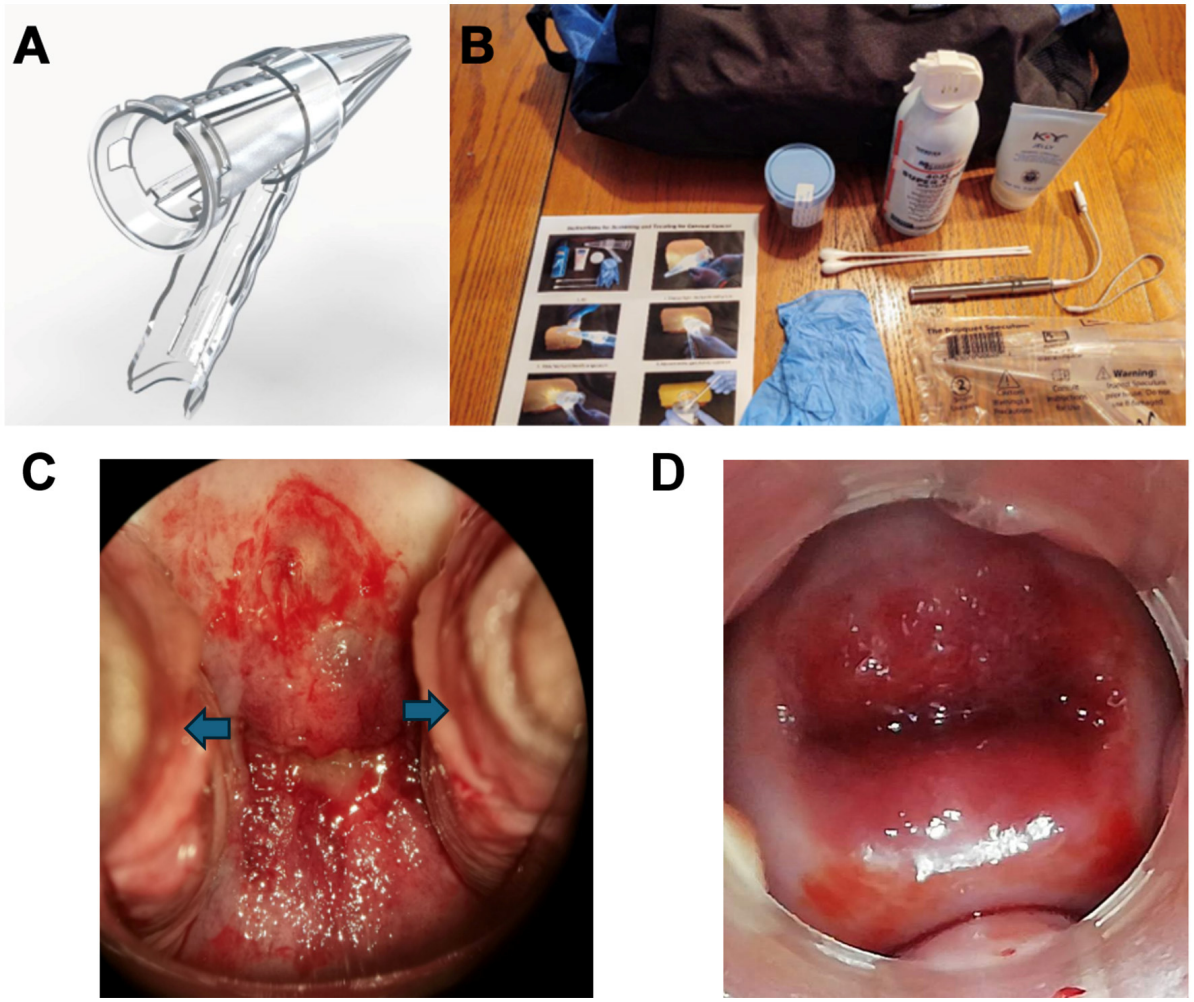
**(A)** The FDA-cleared, CE-marked Bouquet Speculum™. **(B)** Cervical Cancer Cure Kits used in 15 counties in Kenya (2019–2023). **(C)** Incomplete visualization of the cervix with cervical dysplasia noted using a traditional two-bladed speculum with lateral vaginal wall collapse (blue arrows). **(D)** Complete visualization of the cervix with cervical dysplasia noted using the Bouquet Speculum™ without lateral vaginal wall collapse.

A novel vaginal speculum, the Bouquet Speculum™, (Figure 1A) has been proven to provide better visibility of the cervix, is easier to use for the provider and is less uncomfortable for the patient (10, 11). This overcomes some of the limitations of the existing 2-bladed speculum. Better visibility equates to more accurate screening and treatment. Ease-of-use will allow for a more efficient exam by less-experienced providers who may struggle with adequately visualizing the cervix (Figures 1C, D). The new 5-petaled vaginal speculum opens in a radial fashion and distributes the intravaginal forces equally. This results in less discomfort for the patient.

## Screening

In a recent systematic review to create new policy for the W.H.O., it was found that primary HPV screening [DNA probe, Papanicolaou test, and Visual Inspection with Acetic Acid (VIA)] was similarly effective at reducing cervical cancer standardized mortality rates by 63–67% when offered every 5 years. The policy summary concluded that only 9–11% of the population in resource-constrained countries were screened for cervical cancers and precancers (5). Further evaluation of the data revealed that 1.6 billion women out of 2.3 billion women (67%) worldwide, age 20–70, had never been screened for cervical cancer (5).

The current model is based on screening and treating at the point of care. Cervical cancer screening programs using VIA and cryotherapy were conducted by the Governments of Peru, Uganda, and Vietnam in 2011 and 2012. They showed that this was a feasible approach to providing cervical cancer prevention services. Success depended upon mobilizing and educating communities, organizing the services according to women's schedules and needs, and fortifying systems to track clients for follow-up. The sustainability of these screen and treat clinics depends on having an adequate number of trained providers and reducing staff turnover. There were several challenges that were voiced by the patients regarding these screen and treat cervical cancer clinics including distrust of the instruments (introducing infection or causing cervical cancer), travel-related expenses, staff shortages (some women were turned away), “fear of learning the result,” and “fear of pain” (12).

This new model offers a screen, triage, treat and follow up method at the point of care. The screening portion is performed with a more accurate, comfortable, and efficient vaginal speculum and uses the similarly effective VIA method. If acetowhite lesions, indicating cancers or precancers, are seen on the initial screening with acetic acid, the triage portion of the model is then used. This involves the use of a portable colposcope or Smart Phone with AI-assisted cervicography (digital images of the cervix) and real-time interpretation. The software, downloaded onto the Smart Phone can read the digital images in real-time and provide a diagnosis. This increases the sensitivity, specificity, and accuracy of the diagnosis such that overtreatment is reduced. Android AI software has proven accuracy of 97.94%, a sensitivity of 99.05%, and a specificity of 97.16% (13). This allows for more efficient and accurate screening and point of care treatment without the need for reappointments or referral to a tertiary medical center (13–15).

## Treatment

For the providers of the current screen and treat clinics, they were challenged by the subjective nature of interpreting VIA results, inadequate training on VIA, maintaining an adequate supply of materials and compressed gas (CO_2_), repairing non-functioning cryotherapy units, relegating the cryotherapy to physicians only, staff training and turnover, and no standardized referral or follow-up system in place (12).

In this model, once screening is accomplished with VIA, a digital image is evaluated in real- time and determines the diagnosis of the cervical lesion with 97.94% accuracy (13). Cervical intraepithelial neoplasias (CINs) are then treated with cryoablation, when appropriate, at the point of care. The W.H.O. strongly recommends cryotherapy treatment over no treatment for acetowhite lesions when screening with VIA (16). In simple screening clinics with VIA only, with no point of care treatment, many patients do not follow up for definitive treatment (47.2%) as a result of unaffordable transportation cost and limited time to keep the scheduled appointment (17).

The current guidelines from the W.H.O. allow cryoablation treatment with carbon dioxide (CO_2_) and nitrous oxide (N_2_O) that require a temperature of −50° (C). The cryoablation involves a 3-min freeze, 1-min thaw, and a repeat 3-min freeze per the W.H.O. guidelines (18). This temperature allows for cryoablation of the dysplastic cells and stimulates the body's own immune system to help clear the local HPV infection (19). Clinicians have used canister-based (compressed gas) to treat HPV infections of the skin for years (20). It is reasonable to infer that this would be effective on HPV infections of the cervix. Cryoablation surgery is also successfully used for a variety of cancers such as the skin, liver, kidney, bone, lung, prostate, and breast (20).

The compressed gas, tetrafluoroethane, is currently used in products such as Verruca Freeze^®^ for cutaneous lesions, including warts, caused by HPV. It achieves temperatures that are at or lower than the recommended minimal cryosurgical temperatures that were established by the W.H.O. for cervical precancers. It is non-flammable, inexpensive, and portable (21). This overcomes the requirements of bulky and difficult to resource tanks of N_2_O or CO_2_ and expensive ($2,200–2,800 USD) cryosurgical units that can break down or malfunction and then require a referral for treatment (22).

## The cervical cancer cure kit

Due to the inaccessibility, expense, and the challenges mentioned above for these screen and treat clinics, a portable kit and several trained clinicians would allow access to all communities for cervical cancer mass screening, triage, and treatment with canister-based cryogen at the point of care (Figure 1B.) Previous studies using the new speculum and VIA were conducted in Peru, Panama, and Paraguay (23). These kits, with augmented VIA, the new speculum, and canister-based cryogen or LEEP (Loop Electrosurgical Excision Procedure) have been used in 15 out of 47 counties in Kenya since 2019 with 1,888 patients (24). The process is straight forward, and a provider can be trained in <30 min. The first step in this process is the use of the Bouquet Speculum for better visualization of the cervix, and a more efficient and comfortable vaginal speculum exam (10, 11). The next step is applying vinegar (acetic acid) to the cervix and waiting for 45 s. An image is then acquired with either a portable colposcope or a Smart Phone. The image is then analyzed via an app such as Colpadvisor with AI assistance ^1^. Based on the evaluation of the digital images, treatment can be directed at cryoablation (if indicated) or LEEP (if indicated and available).

Used in conjunction with the speculum and cryogen treatment, the kit works to remove limitations to care such as discomfort of screening, inaccurate results, the need to return for treatment, and cost to the patient. Further studies are needed to validate this screen with VIA, triage with Automated Visual Evaluation, treat with canister-based cryogen, and follow up to assess response to treatment. Limitations to this model include expense and coordination of providing the kits for mass screening, insufficient trained personnel, cultural concerns, fears of being screened and treated, and insufficient healthcare literacy.

## Cost comparison

The current cost of screening and treating cervical cancers and precancers in tertiary clinics in Kenya are reported in Table 1 (25, 26). This includes transportation, clinician consultation, and laboratory costs. For screening, VIA at the point of care is the least expensive screening test at $2.00 (USD) per person. This model proposes augmented VIA screening so that lesions can be treated at the point of care. This augmented VIA screening is estimated to cost $5.00 (USD) per woman, which includes the Bouquet Speculum™. For treatment at the Kenyatta National Hospital, the median age of the patient was 41 years, had daily earnings of ~$6.00 (USD) and travel time to the facility averaged 2.8 h (26). The least expensive treatment is $3.00 (USD) for canister-based cryotherapy, if indicated, at the point of care.

**Table 1:** Cost comparison of various screenings (2024) and treatment (2019) of cervical cancer and precancers in Kenya.

**Cost comparison of various screening methods**	
**Screening**	**Average cost (in USD) per person**
Pap smear (clinic)	$41
HPV testing (clinic)	$84
Home-based HPV self-sampling	$82
Colposcopy (clinic)	$65
VIA (point of care; included in Cervical Cancer Cure Kits)	$2
Augmented VIA (point of care; included in Cervical Cancer Cure Kits)	$5
**Cost comparison of various treatments**	
**Treatment**	**Average cost (in USD) per person**
LEEP (Loop electrical excision)	$113
Cryotherapy (standard)	$53
Thermoablation	$60 (estimate)
CKC (Cold knife conization)	$45 (estimate)
Canister-based cryotherapy (included in Cervical Cancer Cure Kits)	$3

## Conclusion

The Cervical Cancer Cure Kits represent a novel, cost-effective, practical, accurate, and sustainable model to screen, triage, and treat cervical cancer and precancers at the point of care in underserved countries and communities. This model may provide an interim solution to the inadequately vaccinated populations throughout the world and help prevent the death of nearly 63 million women over the next 70 years. As the organization, *Doctors Without Borders*, states, “No woman should die of cervical cancer” (27). In summary, this is a simple and inexpensive solution, potentially providing significant global benefits by decreasing morbidity and mortality from cervical cancer and precancers without significant risk for women.

## Data Availability

The original contributions presented in the study are included in the article/supplementary material, further inquiries can be directed to the corresponding authors.

## Footnotes

^1^ https://play.google.com/store/apps/details?id=digital.gynius. colpadvisor

## References

[1] Singh D Vignat J Lorenzoni V Eslahi M Ginsburg O Lauby-Secretan B. Global estimates of incidence and mortality of cervical cancer in 2020: a baseline analysis of the WHO global cervical cancer elimination initiative. Lancet Glob Health. (2022) 11:e197–206. 10.1016/S2214-109X(22)00501-0 36528031 PMC9848409

[2] Chen S Cao Z Prettner K Kuhn M Yang J Jiao L. Estimates and projections of the global economic cost of 29 cancers in 204 countries and territories from 2020 to 2050. JAMA Oncol. (2023) 9:465–72. 10.1001/jamaoncol.2022.7826 36821107 PMC9951101

[3] Nwankwo C Shah R Kwon Y Corman S. Economic and humanistic burden of cervical cancer in the United States. Ann Oncol. (2018) 29:viii350. 10.1093/annonc/mdy285.188 31967943 PMC7307680

[4] Okunade K. Human papillomavirus and cervical cancer. J Obstet Gynaecol. (2019) 40:602–8. 10.1080/01443615.2019.1634030 31500479 PMC7062568

[5] Simms K Keane A Nguyen DT Caruana M Hall MT Lui G. Benefits, harms and cost-effectiveness of cervical screening, triage and treatment strategies for women in the general population. Nat Med. (2023) 29:3050–8. 10.1038/s41591-023-02600-4 38087115 PMC10719104

[6] Ji L Chen M Yao L. Strategies to eliminate cervical cancer in China. Front Oncol. (2023) 13:1105468. 10.3389/fonc.2023.1105468 37333817 PMC10273099

[7] Hall M Simms KT Lew JB Smith MA Brotherton JM Saville M. The projected timeframe until cervical cancer elimination in Australia: a modelling study. Lancet Public Health. (2018) 4:e19–27. 10.1016/S2468-2667(18)30183-X 30291040

[8] Bruni L Serrano B Roura E Alemany L Cowan M Herrero R. Cervical cancer screening programmes and age-specific coverage estimates for 202 countries and territories worldwide: a review and synthetic analysis Lancet Global Health. 10:e1115–27. 10.1016/S2214-109X(22)00241-8 35839811 PMC9296658

[9] Petersen Z Jaca A Ginindza TG Maseko G Takatshana S Ndlovu P. Barriers to uptake of cervical cancer screening services in low-and-middle-income countries: a systematic review. BMC Womens Health. (2022) 22:486. 10.1186/s12905-022-02043-y 36461001 PMC9716693

[10] Bouquet J Naji R Armas CA Roldan V Selkhi S Bentley CZ. An innovative design for the vaginal speculum. Med Devices. (2023) 16:211–8. 10.2147/MDER.S415558 37790696 PMC10542509

[11] Bouquet J Khan S Waters-Kellar L Barsh E Shannon A Clark S. Improving visualization of the cervix, ease of use, and patient comfort using a newly designed vaginal speculum: a pilot study. Med Devices: Evid Res. (2025). 10.2147/MDER.S509134 PMC12211990 40599808

[12] Paul P Winkler JL Bartolini RM Penny ME Huong TT Nga LT. Screen-and-treat approach to cervical cancer prevention using visual inspection with acetic acid and cryotherapy: experiences, perceptions, and beliefs from demonstration projects in Peru, Uganda, and Vietnam. Oncologist. (2013) 18:1278–84. 10.1634/theoncologist.2013-0253 24217554 PMC3868422

[13] Kudva V Prasad K Guruvare S. Android device-based cervical cancer screening for resource-poor settings. J Digit Imaging. (2018) 31:646–54. 10.1007/s10278-018-0083-x 29777323 PMC6148805

[14] Zhao Z Hu B Xu K Jiang Y Xu X Liu Y. A quantitative analysis of artificial intelligence research in cervical cancer: a bibliometric approach utilizing CiteSpace and VOSviewer. Front Oncol. (2024) 14:1431142. 10.3389/fonc.2024.1431142 39296978 PMC11408476

[15] Nakisige C De Fouw M Kabukye J Sultanov M Nazrui N Rahman A. Artificial intelligence and visual inspection in cervical cancer screening. Int J Gynecol Cancer. (2023) 33:1515–21. 10.1136/ijgc-2023-004397 37666527 PMC10579490

[16] WHO Guidelines: Use of Cryotherapy for Cervical Intraepithelial Neoplasia. Geneva: World Health Organization (2011). Available online at: https://www.ncbi.nlm.nih.gov/books/NBK138474/23741775

[17] Ezechi O Petterson KO Gabajabiamila TA Idigbe IE Kuyoro O Ujah IA. Predictors of default from follow-up care in a cervical cancer screening program using direct visual inspection in south-western Nigeria. BMC Health Serv Res. (2014) 14:143. 10.1186/1472-6963-14-143 24678898 PMC3986612

[18] Aarts BM Klompenhouwer EG Rice SL Imani F Baetens T Bex A. Cryoablation and immunotherapy: an overview of evidence on its synergy. Insights Imaging. (2019) 10:53. 10.1186/s13244-019-0727-5 31111237 PMC6527672

[19] Cooper SM Dawber RP. The history of cryosurgery. J R Soc Med. (2001) 4:196–201. 10.1177/014107680109400416 11317629 PMC1281398

[20] Kwak K Yu B Lewandowski RJ Kim DH. Recent progress in cryoablation cancer therapy and nanoparticles mediated cryoablation. Theranostics. (2022) 12:2175–204. 10.7150/thno.67530 35265206 PMC8899563

[21] Verruca Freeze^®^ Material Safety Data Sheet. Rev. (2020). Available online at: https://www.medline.com/media/catalog/Docs/MSDS/MSD_SDSD93384.pdf (accessed October 5, 2024).

[22] Wallach-LL100 Cryosurgical Unit. Available online at: https://mfimedical.com/products/wallach-ll100-cryosurgical-unit (accessed October 5, 2024).

[23] Bouquet J Chernau A McLaughlin R Choudhury Q. A new vaginal speculum and an inexpensive kit to screen and treat dysplasia and cancer of the cervix. J Womens Health Care. (2019) 8:1–3. 10.35248/2167-0420.19.8.470

[24] Kimani D. Cervical Cancer Screening and Treatment in 15/47 Kenyan Counties from 2019–2023. Internal Report.

[25] Africa Health Business. Cost Effectiveness of Cervical Cancer Screening Tests in Kenya. Kenya Report Apr24.pdf. Available online at: https://africahb.com/wp-content/uploads/2024/06/Article_Cost-Effectiveness-of-Cervical-Cancer-Screening-Tests.pdf

[26] Vodicka EL Chung MH Zimmermann MR Kosgei RJ Lee F Mugo NR. Estimating the costs of HIV clinic integrated versus non-integrated treatment of pre-cancerous cervical lesions and costs of cervical cancer treatment in Kenya. PLoS ONE. (2019) 14:e0217331. 10.1371/journal.pone.0217331 31170193 PMC6553698

[27] Doctors Without Borders. No Woman Should Die of Cervical Cancer. The Disease is Preventable, Detectable, and Treatable in the Early Stages (2020). Available online at: https://www.doctorswithoutborders.org/latest/no-woman-should-die-cervical-cancer (accessed October 5, 2024).

